# Microvascular and Structural Alterations of the Macula in Early to Moderate Glaucoma: An Optical Coherence Tomography-Angiography Study

**DOI:** 10.3390/jcm10215017

**Published:** 2021-10-28

**Authors:** Mael Lever, Moritz Glaser, Ying Chen, Christian Halfwassen, Jan Darius Unterlauft, Nikolaos E. Bechrakis, Michael R. R. Böhm

**Affiliations:** 1 Department of Ophthalmology, University Hospital Essen, 45147 Essen, Germany; ying.chen@uk-essen.de (Y.C.); christian.halfwassen@uk-essen.de (C.H.); nikolaos.bechrakis@uk-essen.de (N.E.B.); michael.boehm@uni-due.de (M.R.R.B.); 2 Achim Wessing Institute for Imaging in Ophthalmology, University Hospital Essen, 45147 Essen, Germany; moritz.glaser@uni-due.de; 3 University Hospital of Ophthalmology, Inselspital, 3010 Bern, Switzerland; jandarius.unterlauft@insel.ch

**Keywords:** glaucoma, macula, macular segmentation, optical coherence tomography-angiography, vascular density, blood flow, biomarker

## Abstract

In glaucoma, macular optical coherence tomography (OCT) typically shows a thinning of the three inner segments and OCT-angiography (OCTA) a reduction of the vascular density (VD). It is still unclear if glaucoma directly affects macular VD. This retrospective study included 31 glaucoma patients of early and moderate stage (GS1, GS2, Mills et al.) and 39 healthy individuals. Macular segments’ thickness and superficial and deep plexus vascular density (VD) were obtained using spectral-domain OCT and OCTA, respectively. One-way analysis of variance (ANOVA) was used to compare healthy controls and glaucoma patients according to their glaucoma stage. Using correlation analyses, the association between glaucoma and either OCT or OCTA parameters was evaluated. A glaucoma stage-stratified linear regression analysis was then performed. Inner macular segment and whole retinal thickness were reduced in GS1 and GS2 patients compared to healthy controls (e.g., ganglion cell layer GCL: controls: 47.9 ± 7.4, GS1: 45.8 ± 5.1, GS2: 30.6 ± 9.4, ANOVA: *p* < 0.0001). Regarding OCTA-parameters, the VD of both segmentation levels was reduced in glaucoma patients, particularly when comparing GS2 patients with controls (superficial plexus: *p* = 0.004) and GS2 with GS1 (*p* = 0.0008). Linear regression revealed an association between these parameters and the presence of glaucoma (for superior plexus: R^2^ = 0.059, *p* = 0.043). Finally, a correlation between macular segment thickness and VD was observed, but with a strength increasing with glaucoma severity (GCL and superior plexus VD: controls: R^2^ = 0.23, GS1 R^2^ = 0.40, GS2 R^2^ = 0.76). Despite the glaucoma-independent correlation between macular segment thickness and VD, disease severity strengthens this correlation. This consideration suggests that glaucoma directly influences OCT and OCTA parameters individually.

## 1. Introduction

Over the last two decades, advances in imaging techniques have led to significant changes to the diagnosis and management of ophthalmic conditions. Optical coherence tomography (OCT) is one of the most important of them. In glaucoma, OCT provides precise and objective structural measurements of the optic nerve head (ONH) and of the macula. These data have become crucial to assess the presence and the degree of severity of the disease. OCT measurements were shown to correlate with basic ophthalmologic glaucoma criteria like intraocular pressure (IOP) and ONH cupping, as well as ocular function like visual field perception [1]. While OCT is not recommended as a standalone glaucoma diagnostic tool, it greatly complements more subjective ophthalmologic parameters [2].

Initial research on OCT in glaucoma focused on ONH affection (e.g., peripapillary retinal nerve fiber (RNFL) thickness [3], cupping [4], and Bruch membrane opening [5]). More recent studies also identified macular changes that were useful for the management of glaucoma in adults [6] and children [7]. In particular, the thickness of the three innermost segments of the macula—the nerve fiber layer (NFL), ganglion cell layer (GCL), and inner plexiform layer (IPL), summarized as ganglion cell complex (GCC)—constitutes a reliable biomarker for assessing glaucoma severity and progression [8]. Compared to ONH, the macula presents structural advantages, which reduce possible measurement artifacts and enhance the reliability of the measurements [9]: the macula shows less interindividual variability than the peripapillary RNFL thickness [10], and it lacks blood vessels. These advantages justify the use of macular diagnostic tools for the management of glaucoma patients.

Finally, recent technical improvements to OCT technology led to the development of OCT-angiography (OCTA). In OCTA, up to 100,000 A-scans per second are performed to indirectly detect retinal blood flow [11]. Consecutive computation of the obtained data provides a precise evaluation of retinal and choroidal blood vessel density (VD) without the need for an intravenous dye as in fluoresceine angiography. The obtained three-dimensional representation of the ONH or macula allows for the VD quantification of a superficial and a deep vascular plexus, and measurements of associated parameters such as the surface of the foveal avascular zone (FAZ) [11]. In glaucoma patients, a reduction of the VD was first observed at the ONH [12] and later also at the macula [13]. The following research in glaucoma patients showed that VD quantification using OCTA is reproducible [14], can discriminate between glaucoma and healthy eyes [15,16], and correlates with disease progression [17] and visual function [18].

As already reported, both OCT and OCTA measure different structural characteristics of the macula, and thus a certain correlation between both diagnostic methods has been observed [16]. Yet, the pathophysiological mechanisms that lead to a reduction of VD in glaucoma are still unclear. To help determine if glaucoma has a direct effect on macular VD or indirectly through the reduction of macular thickness, we selected patients with early to moderate glaucoma and measured the alterations of macular segment thickness and OCTA parameters in comparison to healthy adults. These results were then used to analyze the correlation between both diagnostic parameters and to evaluate what individual effect glaucoma has on them.

## 2. Materials and Methods

### 2.1. Study Design

Retrospective chart analysis of patients from whom a macular OCT and OCTA was acquired between May and September 2019 at the Department of Ophthalmology of the University Hospital Essen, Germany. Patients of 18 to 85 years of age were included to the study based on the presence of early glaucoma (defined as stage 1 or stage 2 according to the Mills et al. [19] classification) or the absence of a retinal disease or optic nerve pathology other than glaucoma (healthy controls). Exclusion criteria were a history of ocular trauma or intraocular surgery (excluding uncomplicated cataract surgery), refractive errors > 3 diopters, advanced lens opacity/cataract, the presence of any systemic disease (in particular cardiovascular or neurologic), and current vasoactive systemic medication. Charts lacking data of best corrected visual acuity (BCVA), IOP, anterior segment examination, and/or fundoscopy were also excluded from the study. The eye with the best OCT image quality was selected for further analysis. This study was conducted in accordance with the 1964 Declaration of Helsinki and was approved by the ethics committee of the University Hospital Essen, Germany (approval number: 19-8840-BO).

### 2.2. Data Acquisition

A comprehensive ophthalmic examination was performed, including review of past medical history and current medication, determination of BCVA, slit-lamp examination of the anterior and posterior eye segment, and measurement of IOP (Goldmann applanation tonometer, Haag-Streit, Bern, Switzerland). For glaucoma patients, the examination also included an evaluation of the optic disc linear cup-to-disc ratio (CDR) and stereoscopic ONH photography, the measurement of the central corneal thickness (CCT; TX-20P tonometer, Canon Medical Systems, Zoetermeer, The Netherlands), and a visual field examination using 30-2 static automated perimetry (Twinfield 2, OCULUS Optikgeräte, Wetzlar, Germany).

**Figure 1:**
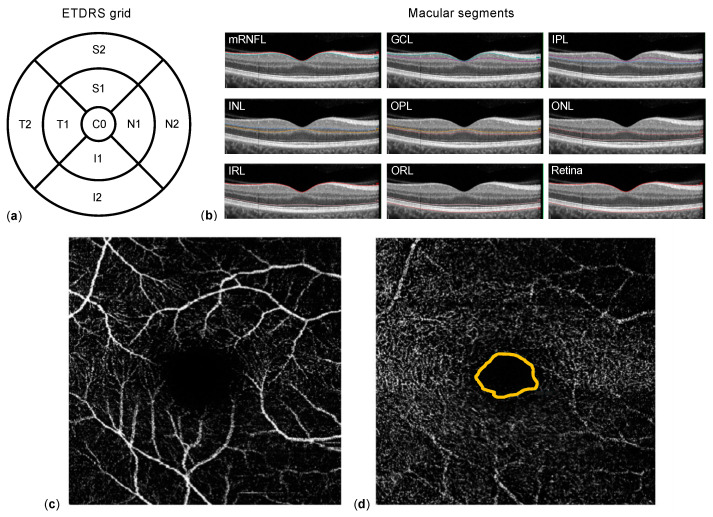
Methodology. (**a**) Representation of the Early Treatment Diabetic Retinopathy Study (ETDRS) grid 1, 2.22, 3.45 mm, which contains nine subfields (C0: center; S1 and S2 superior, N1 and N2 nasal, I1 and I2 inferior, and T1 and T2 temporal) and is used for thickness measurements. The macular segments (**b**) are separated semi-automatically by optical coherence tomography (OCT) software: nerve fiber layer (NFL), ganglion cell layer (GCL), inner plexiform layer (IPL), inner nuclear layer (INL), outer plexiform layer (OPL), outer nuclear layer (ONL)—together inner retinal layers (IRL)—and outer retinal layers (ORL). Example of the superior (**c**) and deep (**d**) vascular plexus of the macula obtained using OCT-angiography; the foveal avascular zone is located within the orange line in (**d**).

Macular spectral domain-OCT and OCTA were obtained using a SPECTRALIS^®^ HRA+OCT (Heidelberg Engineering, Heidelberg, Germany). Corneal curvature values (c-curve) were known for all patients. At least two consecutive examinations of sufficient image quality (quality score ≥ 20) were obtained. For macular OCT, 25 single horizontal axial scans centered to the fovea were acquired. Using the manufacturer’s software, image segmentation was calculated to obtain individual retinal layer thicknesses: total retinal thickness (TRT), nerve fiber layer (NFL), ganglion cell layer (GCL), inner plexiform layer (IPL), inner nuclear layer (INL), outer plexiform layer (OPL), and outer nuclear layer (ONL) (Figure 1c). Additionally, the NFL, GCL, IPL, INL, OPL, and ONL segments were combined as inner retinal layers (IRL). Results of the semi-automated segmentation were inspected and corrected manually if necessary. Thickness results were divided into nine subfields using the 1, 2.22, 3.45 mm grid of the Early Treatment Diabetic Retinopathy Study (ETDRS) (Figure 1b). Thickness values of each subfield were exported using a software plug-in provided by the device manufacturer. For OCTA, the vessel density (VD) of the superficial (SVP) and deep vascular plexus (DVP), as well as the area of the foveal avascular zone (FAZ, in mm^2^), were extracted and analyzed using the ImageJ software (Wayne Rasband, version 1.52e).

### 2.3. Statistical Methods

Numerical data were collected in Microsoft Excel (Microsoft, Redmond, WA, USA). Normal distribution was examined using the D’Agostino and Pearson normality test. Mean values were compared applying Student’s *t*-test, or Mann–Whitney U test, when appropriate. One-way ANOVA was performed to compare multiple subgroups, and the Tukey method was applied for multiple comparison correction in post-hoc analyses. Correlation between parameters was evaluated calculating Pearson or Spearman correlation factors, when appropriate. The statistical analyses were performed using SAS Studio version 3.8 (SAS Institute Inc., Cary, NC, USA) and Prism 9.1 (GraphPad, La Jolla, CA, USA). In the results section of this article, dichotomous variables are presented as absolute and relative frequencies (*n*, %), continuous variables are presented as mean ± standard deviation (SD), and categoric variables as median ± interquartile range (IQR). In general, statistical significance was assumed for *p* < 0.05.

## 3. Results

### 3.1. Patients’ Characteristics

Seventy patients aged 63.0 ± 13.1 years (range: 37–88 years) were included in this study. Age distribution was comparable among glaucoma patients (*n* = 31, 44%) and healthy controls (*n* = 39, 56%). Among the 31 glaucoma patients, disease severity was evaluated as stage 1 according to Mills et al. [19] in 22 cases (71%); stage 2 was identified in nine patients (29%). Male sex was slightly underrepresented in the whole study population (46%). This trend was also similar in the glaucoma and control subgroups. The median BCVA was 0.1 LogMAR (IQR: 0.1); 16% of glaucoma patients had a history of cataract surgery at the time of examination, and this was also the case for 12% of control participants. The overall mean IOP was 13.6 ± 3.0 mm Hg; in the control group, the mean IOP was 14.1 ± 3.2 mm Hg, compared to 12.7 ± 2.0 mm Hg in glaucoma patients. This difference is explained by the antiglaucomatous therapy in the glaucoma group, consisting of a median of 3 topical agents. Epidemiologic data are presented in Table 1.

### 3.2. Differences in Macular Segment Thickness between Glaucoma and Healthy

**Figure 2:**
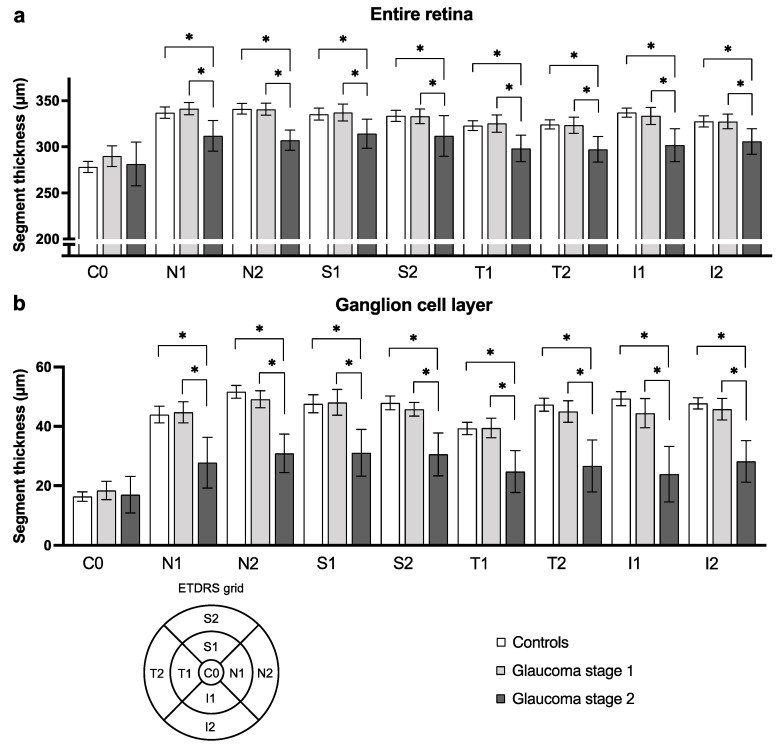
Retina and ganglion cell layer subfields’ thickness differs between glaucoma patients and healthy controls. The figure depicts for exemplary purpose the mean thickness and 95% confidence interval of subfield thickness for the entire retina (**a**) and for the ganglion cell layer (**b**). Statistically significant results of one-way ANOVA comparing each subfield’s mean thickness in patients with glaucoma stage 1, stage 2, and healthy controls are represented (* when *p* < 0.05). Abbreviations: subfields C0, center; S1, inner superior; S2, outer superior; N1, inner nasal; N2, outer nasal; I1, inner inferior; I2, outer inferior; T1, inner temporal; and T2, outer temporal, as shown in the ETDRS grid legend.

First, an analysis of the thickness of the whole macula and its segments was performed for all subfields of the ETDRS grid. To compare results of glaucoma stage 1 and 2 patients and healthy control participants, a one-way ANOVA was performed. The analysis revealed a statistically significant difference between the three groups for the GCL and IPL in all subfields but C0 (e.g., S2 subfield of GCL: controls: 47.9 ± 7.4, GS1: 45.8 ± 5.1, GS2: 30.6 ± 9.4, *p* < 0.0001). A similar observation appeared for measurements of the whole retina and the IRL (e.g., S2 subfield of whole retina: controls: 333.6 ± 18.8, GS1: 333.0 ± 18.0, GS2: 311.8 ± 28.7, *p* = 0.014). The post-hoc analysis returned statistically significant results for the comparison of the control and GS2 groups and of the GS1 and GS2 groups. Even though no such difference was present for the comparison of controls with GS1, a constant trend of GS1 patients having a slightly thinner GCL, IPL, IRL, and whole retina is visible. For measurements of the NFL, INL, OPL and ONL, as well as for ORL, no such difference could be discerned in our cohort (data not shown). Table 2 presents an excerpt of these measurements and ANOVA results. A graphical presentation of the results for the whole retina and for GCL is provided in Figure 2.

### 3.3. Macular Vasculature Differences between Glaucoma Patients and Healthy Controls

**Figure 3:**
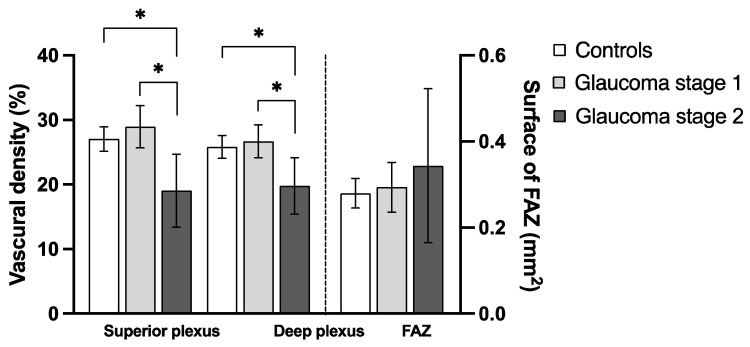
Superficial and deep vascular density of the macula differ between glaucoma patients and controls. This figure shows the comparison (mean and 95% confidence interval) of optical coherence tomography-angiography (OCTA) parameters between healthy controls (white bars) and glaucoma patients of stage 1 (light grey) and 2 (dark grey). Statistically significant results (*p* < 0.05) of one-way ANOVA are marked with an asterisk *. Abbreviation: FAZ, foveal avascular zone.

Regarding the macular vascular parameters provided by OCTA, no significant difference appeared when comparing all glaucoma patients and control group (Table 3). However, the separate analysis of stage 1 and stage 2 glaucoma patients using one-way ANOVA returned statistical differences between the three groups. This was apparent for the vascular density (VD) of the superficial plexus (*p* = 0.0011) and of the deep plexus (*p* = 0.0072) (Table 4). The additional post-hoc analysis revealed a difference predominantly between controls and the GS2 group as well as between the GS1 and GS2 groups, but not between controls and GS1 (Figure 3).

To evaluate if OCTA parameters correlate with the presence of glaucoma, we calculated the Spearman correlation factor between OCTA parameters and glaucoma stage. For all three parameters—FAZ area and VD of the superficial and deep plexus—no correlation was detected (data not shown). Further, we performed univariate linear regression analysis, which returned a weak association between the glaucoma stage and VD of both plexuses (superior plexus: R^2^ = 0.059, deep plexus: R^2^ = 0.058) (Table 5).

### 3.4. Correlation of Vascular Density and Macular Segment Thickness

Finally, to evaluate the strength of the association between macular segment thickness and VD of the superficial and deep plexuses, a correlation analysis between all subfields of all macular segments and the vascular density of the superior and deep plexus was performed. The parameters with the highest correlation factor in the entire cohort and in glaucoma stage 2 patients were selected for further regression modelling. Regarding the superior plexus, the T2 subfield of the GCL was selected (entire cohort: Pearson r = 0.67, 95% CI: 0.52–0.78; GS2: Pearson r = 0.87, 95% CI: 0.50–0.97). Regarding the deep plexus, the N2 subfield of IPL showed the highest correlation in both groups (entire cohort: Pearson r = 0.45, 95% CI: 0.24–0.62; GS2: Pearson r = 0.82, 95% CI: 0.33–0.96). A univariate linear regression analysis was performed using the selected parameter as independent variable. This analysis showed that the strengths of association between GCL thickness (T2 subfield) and vascular density increases with glaucoma severity. According to our results, an intermediate association is detectable in controls (R^2^ = 0.23), as well as in GS1 patients (R^2^ = 0.40), and a strong association in GS2 patients (R^2^ = 0.76). Similarly, the strengths of association between IPL thickness (N2 subfield) and glaucoma increase with disease severity: R^2^ is low — shows no association — in controls (R^2^ = 0.027) and in GS1 patients (R^2^ = 0.097) but high – shows a strong association – in GS2 patients (R^2^ = 0.66). These results are presented in Table 6.

## 4. Discussion

The present study investigates the relation between macular segments’ thickness as well as OCTA-assessed vascular density in the context of early or moderate glaucoma. The main findings of the study are:

- The vascular density of the deep and superficial plexus is reduced in glaucoma and correlates with the presence of glaucoma.
- Differences in macular vascular density are mainly detectable in GS2 but less so in GS1 eyes.
- The foveal avascular zone is not affected by glaucoma.
- Glaucoma severity directly influences the strength of association between macular inner segments’ thickness and vascular density.

The study population analyzed in the present work is composed of healthy participants and patients with early or moderate glaucoma (as defined by Mills et al. [19]). Adult participants of a wide age range of 37 to 88 years were included, and the gender distribution was comparable between all subgroups. A comparison of the macular segments’ thickness was performed between healthy controls and glaucoma patients, and between glaucoma stages. The present analysis showed a reduction of inner macular segment thickness (the GCL and IPL), in all ETDRS subfields except C0. This observation is consistent with recent reports about the glaucoma-induced loss of retinal ganglion cells located in the inner retinal layers [20] and the subsequent thinning of these layers. Similar observations were made in both adults [16,21] and children [7]. While macular diagnostics in glaucoma focuses mainly on the NFL, GCL, and IPL thickness (summarized as GCC, [21]), the presented one-way ANOVA also showed a thickness reduction of the entire retinal thickness and of the inner retinal layers (IRL). Additionally, the post-hoc analysis revealed a trend towards a thinning of the GCL and IPL between GS1 patients and controls. This difference is statistically significant between GS2 patients and controls as well as between GS2 and GS1 patients. These observations are in line with previous work stating that structural macular deterioration occurs early in glaucoma [22], even prior to detectable functional (perimetric) damage [23].

In most open-angle glaucoma patients, the glaucoma-induced structural retinal changes are explained by elevated IOP [24]. In addition to this, a vascular etiology for glaucoma progression was proposed [25]. This hypothesis is fueled by the possible occurrence and progression of glaucoma despite a seemingly acceptable IOP, which is qualified as normal tension glaucoma (NTG). The dysregulation of ocular blood flow observed in NTG patients is thought to be their leading pathophysiological mechanism [26]. Ocular blood flow monitoring never gained clinical relevance due to practicability reasons, high interindividual variability, and a lack of reproducible quantitative measurement methods. This trend changed with the development of OCT-angiography, which allows for a quantitative and objective, dye-free, and non-invasive method to measure blood flow at the optic nerve head [12]. Later, vascular density changes in glaucoma patients were also described at the macula [13]. The present study showed macular VD differences between early to moderate glaucoma patients and healthy individuals. Our analyses showed a clear reduction of VD in superficial and deep macular plexuses in GS2 patients compared to both controls and GS1 patients. While it may be unexpected that controls and GS1 patients show a similar VD, this lack of statistical difference could be explained by the relatively high mean age in our cohort or by a number of participants too low to detect a slight difference between both subgroups. Overall, our observations are in line with recent publications: while early studies described the decrease of the superficial plexus VD in glaucoma patients [13,15], newer investigations observed additional differences in the deep plexus [17,27]. The provided additional linear regression analyses returned a weak but statistically significant association between vascular density of both superficial and deep macular plexuses and glaucoma stage (no glaucoma, GS1, and GS2). This observation is similar to recent works studying the glaucoma diagnostic ability of macular OCTA measurements [16,28].

When studying macular OCTA parameters, FAZ area in our study population was also analyzed. FAZ area is known to increase in retinopathies involving ischemia (e.g., diabetic retinopathy) [29,30]. However, this does not seem to be the case in glaucoma. Accordingly, our results show no difference in FAZ size between healthy controls and glaucoma patients regardless of the disease stage. This was also observed recently by Lommatzsch et al. [31] for glaucoma patients with peripheral visual field defects, whereas patients with central VF defects (not present in our study) had an enlarged FAZ. This can be explained by the fact that the fovea only consists of photoreceptors, which are usually not directly affected in glaucoma. Additionally, the lacking difference between FAZ area in glaucomatous and healthy eyes is comparable to the lacking difference of C0 ETDRS-subfield thickness between these groups. In summary, the FAZ does not seem to be an informative biomarker for quantifying glaucomatous damage.

In the present study population, inner macular segment thickness and VD are both reduced in glaucoma patients compared the healthy controls. The fact that glaucoma leads to a reduced GCC thickness is well recognized [8], and a comparable effect of glaucoma on macular VD was also observed in the present study (Table 5) and described previously [18]. As these parameters both characterize a different aspect of the same anatomic region (i.e., the macula), it could be possible that glaucoma does not affect macular thickness and VD independently but rather influence one parameter (e.g., VD), leading to an indirect affection of the other parameter (e.g., macular thickness). To verify this hypothesis, a glaucoma stage-stratified quantification of the association between VD and inner macular thickness was performed. A notable strength of the present study population is its reduction of other potential confounders for macular parameters: the age and sex distribution are highly consistent between the control and glaucoma groups, and comorbidities knowingly altering retinal blood flow or macular thickness measurements by OCT(A) were excluded. Our analysis of the whole cohort returned a moderate correlation between GCL and IPL, and the macular VD. This suggests that a reduced VD correlates with a reduced macular segment thickness (and vice versa), which has been described previously in POAG [16] and NTG patients [18]. However, the glaucoma stage-stratified analysis revealed a stronger correlation between inner segment thickness and macular VD in GS2 patients than in GS1 patients or healthy controls: the goodness-of-fit of linear regression (R^2^) increased along with disease severity. This allows one to postulate that glaucoma is causing a reduction of macular inner segment thickness and vascular density in addition to the physiological association between these two parameters. It is still unclear whether RGC loss (e.g., inner macular segment thinning) is the cause or the consequence of vascular density reduction, but the present analysis adds up to the common assumption that glaucoma severity is strongly associated with both inner macular thickness and VD.

The present study has several limitations that need to be discussed. First, it is unclear how topic antiglaucomatous medication affects ocular blood flow and OCTA measurements, which weakens the validity of comparisons of glaucoma patients and medication-naïve controls. Similarly, a possible vasoactive effect of phenylephrine used for pupil dilatation prior to OCTA measurements could also not be ruled out. Further, it must be noted that segmentation differences between devices of different manufacturers can affect the comparability of the present results with other studies. Another aspect influencing results reproducibility is the present use of 3 × 3 mm scans, as other scan sizes (6 × 6 mm or even 9 × 9 mm) might return different results. Finally, the relatively small size of the cohort analyzed here, the focus on early to moderate glaucoma, and absence of preperimetric patients do not allow for generalization of the present results, particularly for advanced glaucoma stages.

## 5. Conclusions

Using OCT and OCTA to identify and monitor the structural and vascular changes at the macula in glaucoma patients is gaining in clinical relevance. In the present study, an inner macular segment thinning is present in early to moderate glaucoma. Regarding OCTA-parameters, the vascular density of the superficial and deep plexuses is reduced compared to healthy controls, whereas no difference of the foveal avascular zone area is visible. Finally, a correlation between inner macular segment thickness and superficial and deep plexus vascular density is already present in healthy individuals. However, the strength of this association seems to be directly influenced by glaucoma severity, suggesting that glaucoma directly affects both diagnostic parameters.

## Figures and Tables

**Table 1:** Epidemiologic and general ophthalmologic characteristics of patients.

Parameter	Value	*p*-Value ^1^
Patients (*n*)	70	
Sex (male:female % (*n*))	46:54% (32:38)	
Glaucoma	45:55% (14:17)	
Healthy	46:54% (18:21)	
Eye (right:left % (*n*))	56:44% (39:31)	
Diagnosis (glaucoma: healthy % (*n*))	44:56% (31:39)	
Stage 1 (% of glaucoma (*n*))	71.0% (22)	
Stage 2 (% of glaucoma (*n*))	29.0% (9)	
Age (mean ± SD (y))	63.0 ± 13.1	
Glaucoma	63.8 ± 14.0	0.64
Glaucoma stage 1	64.7 ± 14.8	0.52
Glaucoma stage 2	61.8 ± 12.5	0.90
Healthy	62.4 ± 12.5	
BCVA (median ± IQR (LogMar))	0.1 ± 0.1	
Glaucoma	0.1 ± 0.2	0.23
Healthy	0.0 ± 0.1	
IOP (mean ± SD (mm Hg))	13.6 ± 3.0	
Glaucoma	12.7 ± 2.0	0.066
Healthy	14.1 ± 3.2	
Linear CDR * (median ± IQR)	0.7 ± 0.3	
Perimetry (MD) * (mean ± SD (dB))	2.0 ± 4.3	

^1^ *p*-values of *t*-test (age, IOP, and BCVA) between the healthy controls and the respective glaucoma group. * Parameters displaying only data of the glaucoma group. Abbreviations: BCVA: best corrected visual acuity; IOP: intraocular pressure; CDR: cup-to-disc ratio (by fundoscopy and/or ONH photography); y: years; MD: mean deviation; dB: decibel; SD: standard deviation; and IQR: interquartile range.

**Table 2:** Macular segment thickness differs between glaucoma patients and healthy controls. The table presents the mean thickness of selected macular segment sectors and the results of one-way ANOVA comparing thickness of each subfield in patients with glaucoma stage 1, stage 2, and healthy controls. The comparison of controls and glaucoma stage 1 patients almost never returned a statistically significant difference and thus is not presented here.

Macular Segment	Mean Thickness (µm) ± SD			ANOVA Summary		Adjusted *p*-Value	
	Controls	Glaucoma Stage 1 (GS1)	Glaucoma Stage 2 (GS2)	F	*p*-Value	Controls vs. GS2	GS1 vs. GS2
Retina							
Inner superior (S1)	335.4 ± 20.0	337.2 ± 21.0	314.2 ± 20.5	4.54	0.014	0.018	0.016
Outer superior (S2)	333.6 ± 18.8	333.0 ± 18.0	311.8 ± 28.7	4.59	0.014	0.012	0.024
Inner inferior (I1)	337.0 ± 14.9	333.5 ± 20.9	301.8 ± 23.1	14.1	<0.0001	<0.0001	0.0001
Outer inferior (I2)	327.6 ± 8.7	327.5 ± 17.9	305.7 ± 17.9	5.57	0.0058	0.0054	0.010
Ganglion cell layer							
Inner superior (S1)	47.6 ± 9.6	48.1 ± 9.8	31.1 ± 10.3	11.8	<0.0001	<0.0001	<0.0001
Outer superior (S2)	47.9 ± 7.4	45.8 ± 5.1	30.6 ± 9.4	23.3	<0.0001	<0.0001	<0.0001
Inner inferior (I1)	49.4 ± 7.2	44.5 ± 11.1	23.9 ± 12.2	27.7	<0.0001	<0.0001	<0.0001
Outer inferior (I2)	47.7 ± 5.9	45.8 ± 8.2	28.2 ± 9.1	28.1	<0.0001	<0.0001	<0.0001
Inner plexiform layer							
Inner superior (S1)	39.8 ± 5.5	39.8 ± 5.1	30.1 ± 6.8	11.9	<0.0001	<0.0001	0.0001
Outer superior (S2)	38.3 ± 4.8	37.3 ± 4.1	27.7 ± 7.3	16.8	<0.0001	<0.0001	<0.0001
Inner inferior (I1)	40.3 ± 4.2	38.0 ± 6.1	27.4 ± 8.3	19.8	<0.0001	<0.0001	<0.0001
Outer inferior (I2)	37.7 ± 4.4	37.2 ± 5.2	27.2 ± 5.4	18.5	<0.0001	<0.0001	<0.0001

*p*-values of ANOVA (analysis of variance). Abbreviation: SD, standard deviation.

**Table 3:** OCTA parameters in glaucoma patients and healthy controls. The table displays the mean ± standard deviation and Students *t*-test (for SVP and DVP) and Mann–Whitney U results (for FAZ) of the foveal avascular zone area (FAZ) and vascular density (VD) of the macular superficial (SVP) and deep plexus (DVP) in glaucoma patients and healthy controls.

	Controls	Glaucoma	*p*-Value	Glaucoma Stage 1	Glaucoma Stage 2
FAZ area (mm^2^)	0.28 ± 0.11	0.31 ± 0.16	0.37	0.29 ± 0.13	0.34 ± 0.23
Superior plexus VD (%)	27.0 ± 5.8	26.1 ± 8.6	0.58	29.0 ± 7.4	19.0 ± 7.4
Deep plexus VD (%)	25.8 ± 5.4	24.7 ± 6.4	0.42	26.7 ± 5.7	19.8 ± 5.7

Abbreviations: OCTA: optical coherence tomography-angiography.

**Table 4:** Summary of one-way ANOVA results for OCTA parameters between glaucoma patients and healthy controls. The table displays results of the one-way analysis of variance (ANOVA) comparing optical coherence tomography-angiography (OCTA) parameters of healthy controls and glaucoma patients of stage 1 (GS1) and stage 2 (GS2).

	F of ANOVA	*p*-Value	Mean Difference	95% CI	Adjusted *p*-Value
FAZ (mm^2^)	0.84	0.44			
Controls vs. GS1			−0.015	−0.10 to 0.071	0.91
Controls vs. GS2			−0.064	−0.18 to 0.055	0.40
GS1 vs. GS2			−0.050	−0.18 to 0.078	0.62
Superior plexus (%)	7.8	**0.0011**			
Controls vs. GS1			−1.9	−6.1 to 2.3	0.52
Controls vs. GS2			8.0	2.2 to 13.8	**0.0040**
GS1 vs. GS2			9.9	3.8 to 16.1	**0.0008**
Deep plexus (%)	5.3	**0.0072**			
Controls vs. GS1			−0.85	−4.4 to 2.7	0.83
Controls vs. GS2			6.1	1.1 to 11.0	**0.0120**
GS1 vs. GS2			6.9	1.6 to 12.2	**0.0069**

*p*-Values are bold and underlined when statistically significant (*p* < 0.05). Abbreviation: FAZ: foveal avascular zone, 95% CI: 95% confidence interval of the mean difference.

**Table 5:** Linear regression analysis between OCTA parameters and glaucoma stage. The table displays the results of univariate linear regression between the glaucoma stage and optical coherence tomography-angiography (OCTA) parameters: foveal avascular zone surface (FAZ) and vascular density of the macular superficial and deep plexus.

	Parameter Estimate	95% CI	R^2^	*p*-Value
FAZ	0.78	−0.50 to 2.1	0.021	0.23
Superior plexus	−0.024	−0.048 to −0.00079	0.059	**0.043**
Deep plexus	−0.029	−0.058 to −0.00075	0.058	**0.045**

*p*-Values are bold and underlined when statistically significant (*p* < 0.05). Abbreviations: FAZ: foveal avascular zone, 95% CI: 95% confidence interval of the parameter estimate, and R^2^: Tjur’s pseudo R^2^.

**Table 6:** Correlation between vascular density and macular segment thickness is strongest in glaucoma patients. The table presents the results of univariate linear regression analysis between either the outer temporal (T2) subfield of the ganglion cell layer (GCL) and the VD of the superficial plexus, or between the outer nasal (N2) subfield of the inner plexiform layer (IPL) and the VD of the deep plexus in optical coherence tomography-angiography.

	Parameter Estimate	95% CI	R^2^
T2 of GCL → superficial plexus vascular density			
Controls	0.41	0.16 to 0.66	0.23
Glaucoma stage 1	0.57	0.24 to 0.89	0.40
Glaucoma stage 2	0.56	0.28 to 0.84	0.76
N2 of IPL → deep plexus vascular density			
Controls	0.19	−0.19 to 0.57	0.027
Glaucoma stage 1	0.43	−0.18 to 1.0	0.097
Glaucoma stage 2	0.74	0.27 to 1.0	0.66

Abbreviation: 95% CI, 95% confidence interval.

## Data Availability

The data supporting the reported results can be provided by the corresponding author on reasonable request.
